# Medulloblastoma has a global impact on health related quality of life: Findings from an international cohort

**DOI:** 10.1002/cam4.2701

**Published:** 2019-11-21

**Authors:** Cynthia B. de Medeiros, Iska Moxon‐Emre, Nadia Scantlebury, David Malkin, Vijay Ramaswamy, Alexandra Decker, Nicole Law, Toshihiro Kumabe, Jeffrey Leonard, Josh Rubin, Shin Jung, Seung‐Ki Kim, Nalin Gupta, William Weiss, Claudia C. Faria, Rajeev Vibhakar, Lucie Lafay‐Cousin, Jennifer Chan, Johan M. Kros, Laura Janzen, Michael D. Taylor, Eric Bouffet, Donald J. Mabbott

**Affiliations:** ^1^ Department of Psychology The Hospital for Sick Children Toronto ON Canada; ^2^ Neurosciences and Mental Health The Hospital for Sick Children Toronto ON Canada; ^3^ Department of Psychology The University of Toronto Toronto ON Canada; ^4^ Pediatric Oncology Group of Ontario Toronto ON Canada; ^5^ Department of Hematology/Oncology The Hospital for Sick Children Toronto ON Canada; ^6^ Department of Pediatrics University of Toronto Toronto ON Canada; ^7^ Tohoku University Graduate School of Medicine Sendai Japan; ^8^ Nationwide Children's Hospital Columbus OH USA; ^9^ St. Louis Children's Hospital St. Louis MO USA; ^10^ Chonnam National University Hwasun‐gun Korea; ^11^ Seoul National University Hospital Seoul Korea; ^12^ University of California San Francisco San Francisco CA USA; ^13^ Hospital de Santa Maria Centro Hospitalar Lisboa Norte Lisbon Portugal; ^14^ University of Colorado Denver Aurora CO USA; ^15^ Alberta Children's Hospital Calgary AB Canada; ^16^ University of Calgary Calgary AB Canada; ^17^ Erasmus Medical Center Rotterdam The Netherlands; ^18^ Developmental and Stem Cell Biology Program The Hospital for Sick Children Toronto ON Canada; ^19^ The Arthur and Sonia Labatt Brain Tumour Research Centre The Hospital for Sick Children Toronto ON Canada; ^20^ Division of Neurosurgery The Hospital for Sick Children Toronto ON Canada; ^21^ Department of Surgery Department of Laboratory Medicine and Pathobiology and Department of Medical Biophysics University of Toronto Toronto ON Canada

**Keywords:** development, medulloblastoma, pediatric psychology, quality of life, survivors of childhood cancer

## Abstract

**Background:**

Understanding the global impact of medulloblastoma on health related quality of life (HRQL) is critical to characterizing the broad impact of this disease and realizing the benefits of modern treatments. We evaluated HRQL in an international cohort of pediatric medulloblastoma patients.

**Methods:**

Seventy‐six patients were selected from 10 sites across North America, Europe, and Asia, who participated in the Medulloblastoma Advanced Genomics International Consortium (MAGIC). The Health Utilities Index (HUI) was administered to patients and/or parents at each site. Responses were used to determine overall HRQL and attributes (ie specific subdomains). The impact of various demographic and medical variables on HRQL was considered—including molecular subgroup.

**Results:**

The majority of patients reported having moderate or severe overall burden of morbidity for both the HUI2 and HUI3 (HUI2 = 60%; HUI3 = 72.1%) when proxy‐assessed. Self‐care in the HUI2 was rated as higher (ie better outcome) for patients from Western versus Eastern sites, *P* = .02. Patients with nonmetastatic status had higher values (ie better outcomes) for the HUI3 hearing, HUI3 pain, and HUI2 pain, all *P* < .05. Patients treated with a gross total resection also had better outcomes for the HUI3 hearing (*P* = .04). However, those who underwent a gross total resection reported having worse outcomes on the HUI3 vision (*P* = .02). No differences in HRQL were evident as a function of subgroup.

**Conclusions:**

By examining an international sample of survivors, we characterized the worldwide impact of medulloblastoma. This is a critical first step in developing global standards for evaluating long‐term outcomes.

## Graphical abstract

Despite the significant advances in our knowledge of medulloblastoma and the resulting advantages of improved therapy, health related quality of life (HRQL) requires further attention globally. Assessing HRQL in an international sample, we found that the majority of patients reported having moderate or severe overall burden of morbidity. This demonstrates the need for the development of universal standards for assessing HRQL through international collaboration in order to improve the lives of all pediatric medulloblastoma survivors.

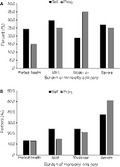

## 1 INTRODUCTION

Medulloblastoma accounts for 20%‐25% of all pediatric brain tumors in high income countries, but has a global impact on children's overall health.^1^ Survival rates for medulloblastoma have increased significantly due to improved treatment methods, with five year survival rates that range from 70% to 85%.^2^, ^3^, ^4^, ^5^ However, there is a disparity in survival rates in low to middle income countries—ranging from 33% to 73%.^6^, ^7^ Social, cognitive, and neurological long‐term late effects have the potential to compromise the ongoing quality of life of survivors worldwide.^8^, ^9^, ^10^, ^11^, ^12^ Despite this global impact—there is a dearth of internationally collaborative studies examining long‐term outcomes in this vulnerable population. International studies investigating medulloblastoma primarily focus on improving treatment protocols, however, even such studies demonstrate challenges in recruiting sufficient patients across multiple sites.^13^ Such studies are critical for characterizing the impact of this disease on health related quality of life (HRQL) and to implement the benefits of new knowledge about medulloblastoma across the world.

Previous studies on HRQL of brain tumor survivors have been limited to heterogeneous cohorts with multiple diagnoses and patients within the same continent (eg North America, Europe).^14^, ^15^ HRQL of medulloblastoma survivors has been examined using patients within a single country.^8^, ^16^ An Italian cohort of medulloblastoma survivors displayed lower HRQL compared to those diagnosed with astrocytoma or a nontumor group.^16^ In a multicenter South Korean cohort, age at diagnosis for pediatric medulloblastoma survivors did not predict HRQL.^8^ Considering the worldwide impact of medulloblastoma, it would be beneficial to investigate HRQL across an international sample.

International collaboration has played an important role in advancing our understanding of the molecular diversity of medulloblastoma. In particular, data obtained from large international cohort studies, consensus statements, and meta‐analyses have led to the identification of four distinct molecular subgroups of medulloblastoma: sonic hedgehog (SHH), Group 4, Group 3, and wingless (WNT).^17^, ^18^, ^19^, ^20^ Considering the long‐term negative effects of treatment, individualizing treatment based on molecular subgroup requires a balance between survival and HRQL. Similarly, an international focus on HRQL of survivors of pediatric medulloblastoma is important to realize the clinical impact of molecular subgroups. Since the total number of childhood medulloblastoma survivors is relatively small, international collaboration is crucial to obtain sufficiently large sample sizes to accurately assess the impact of medulloblastoma on HRQL. When doing so, it is important to consider international differences and specific disease factors, particularly subgroup status, on functional outcome.

Here, we examined HRQL—for the first time in a multi‐continental cohort of pediatric medulloblastoma survivors—including survivors from North America, Europe, and Asia. We used the Health Utilities Index (HUI) (© Health Utilities Inc)^21^, ^22^ as it is a widely used and well‐validated measure of HRQL^22^ that has been employed in studies of childhood brain tumor survivors.^23^, ^24^, ^25^ The HUI provides scores for functional attributes including cognition, pain, and emotion, which are then aggregated to provide a score for overall burden of morbidity, a measure of the impact that the disease (ie medulloblastoma) has on overall HRQL. Most importantly, the HUI has been translated and administered in different languages, including English, Japanese, Korean, Portuguese and Dutch.^22^

Since HRQL has never been characterized in an international sample of pediatric medulloblastoma survivors, our goal was to understand how subgroup and medical and demographic variables impact HRQL in this population. By understanding how these factors impact HRQL, ultimately, this information can be used to determine if therapies should be modified for specific subgroups to improve HRQL without dramatically changing their prognosis.

## 2 MATERIALS AND METHODS

Seventy‐six children with pathologic confirmation of medulloblastoma participated in the study (SHH, n = 16; Group 4, n = 34; Group 3, n = 15; and WNT, n = 7). Subgroup information was unavailable for four patients. The Medulloblastoma Advanced Genomics International Consortium (MAGIC) tumor bank holds over 2000 frozen medulloblastomas from more than 90 high quality pediatric neuro‐oncology centers from around the world. Of these centers, 34 were approached and contacted via email. Twenty‐one replied expressing interest in participating. Of those, ten obtained local ethics approval and provided data (Canada (n = 31): Toronto and Calgary; USA (n = 15): St. Louis, San Francisco, Columbus, and Aurora; Japan (n = 12): Sendai; South Korea (n = 13): Chonnam and Seoul; Portugal (n = 2): Lisbon; and the Netherlands (n = 3): Rotterdam). Data was not received from the remaining sites despite obtaining ethics approval, because data was not received within the time frame required for the study (n = 5) or the site was lost to follow‐up contact (n = 6). Each participating site identified eligible patients based on the following inclusion criteria (a) diagnosed with a medulloblastoma between August 1995 and August 2010 and (b) tissue sample is included in the MAGIC tissue bank. All participating sites obtained research ethics approval from their respective institutional boards and conformed to the ethical standards according to the Declaration of Helsinki.

Parents/guardians were included only if their child qualified. Patients were excluded if (a) they were diagnosed with a medulloblastoma prior to August 1995 or after August 2010, or (b) their tissue sample was not included in the MAGIC tissue bank. Eligible participants were approached about the study during one of their hospital visits. Informed consent (and assent, where applicable) was obtained at each site prior to patients (and/or parent(s)/legal guardian(s)) completing the HUI. The HUI was completed at a single time point by each participant.

Demographic and medical features of the entire sample, by region and subgroup are summarized in Table 1. Patients treated with craniospinal irradiation (CSI) received either standard‐ (ie, 30.6 to 39.4 Gy) or reduced‐dose (ie, 18.0 to 23.4 Gy) radiation to the entire brain and spine with a boost to the posterior fossa or the primary tumor bed.

**Table 1:** (a) Medical and demographic variables for the entire sample, by region (Western [Europe and North America] vs Eastern [Asia] sites), and subgroup (SHH, Group 4, Group 3 and WNT). (b) Missing medical and demographic variables for the entire sample, by region (Western [Europe and North America] vs Eastern [Asia] sites), and subgroup

(a) Medical and demographic variable	Entire sample n = 76	Western sample n = 51	Eastern sample n = 25	SHH n = 16	Group 4 n = 34	Group 3 n = 15	WNT n = 7
Age at diagnosis (years)							
Mean (SD)	6.71 (3.56)	6.82 (3.41)	6.48 (3.9)	4.65 (3.60)	7.62 (3.18)	6.52 (4.05)	8.28 (2.45)
Range	0.33‐17.0	0.33‐14.95	1.0‐17.0	0.33‐14.33	3.04‐14.95	2.0‐17.0	5.81‐12.0
Time since diagnosis (years)							
Mean (SD)	6.58 (4.00)	6.31 (4.14)	7.13 (3.74)	8.13 (4.05)	6.41 (3.84)	6.76 (4.85)	4.87 (2.23)
Range	0.54‐17.67	0.92‐17.67	0.54‐15.91	2.67‐15.91	0.54‐16.51	1.24‐17.67	0.92‐6.88
Sex (% male)	70.7	68.0	76.0	62.5	67.6	86.7	57.1
Metastatic status (% M+)	25.3	28.0	20.0	18.8	29.4	20.0	42.9
Gross total resection (%)	85.1	79.6	88.0	93.8	75.8	93.3	85.7
CSI (%)							
Standard dose	31.5	40.8	12.5	25.0	30.3	35.7	42.9
Reduced dose	65.8	57.1	83.3	62.5	69.7	64.3	57.1
None	2.7	2.0	4.2	12.5	0.0	0.0	0.0
Chemotherapy (%)	98.7	98.0	100.0	93.8	100.0	100.0	100.0

(b) Missing medical and demographic variable	Entire sample	Western sample	Eastern sample	SHH	Group 4	Group 3	WNT
Age at diagnosis	1	1	0	0	0	0	0
Time since diagnosis	2	2	0	0	1	0	0
Sex	1	1	0	0	0	0	0
Metastatic status	1	1	0	0	0	0	0
Resection	2	2	0	0	1	0	0
CSI	3	2	1	0	1	1	0
Chemotherapy	1	1	0	0	0	0	0
Subgroup	4	4	0	N/A	N/A	N/A	N/A

Abbreviations: M+, metastatic; SD, standard deviation; SHH, sonic hedgehog; WNT, wingless.

### 2.1 Health Utilities Index

HRQL was evaluated using the 15‐item questionnaire version of the HUI. Results from the HUI can be tabulated to derive scores for two complementary systems: HUI Mark 2 (HUI2)^26^ and HUI Mark 3 (HUI3).^27^ Respondents were asked to respond to the questions based on the patient's “usual” health status. At each site, the HUI was self‐administered and completed by proxy‐ (parent/guardian) (n = 36) and/or self‐assessed (for patients 12 years of age or older and with capacity; n = 13). Whenever possible, both proxy‐ and self‐assessed versions were completed (n = 27). Relevant translations of the HUI were employed for each site, including either English, Dutch, Japanese, Portuguese, or Korean. Responses from the HUI were used to provide attribute levels and utility scores.

### 2.2 Scoring of the HUI

Responses to the HUI were used to determine attribute levels and single‐attribute utility scores for the HUI3, then the HUI2, as some of the HUI3 attribute levels and utility scores are required to obtain scores for the HUI2. The HUI attribute levels and HUI single‐attribute utility scores are not intended to provide clinical significance at the individual level, nor are there normative data associated with these scores. Rather, the HUI attribute levels and single‐attribute utility scores reflect functional classes of disability. The HUI3 has eight attributes: (a) vision, (b) hearing, (c) speech, (d) emotion, (e) pain, (f) ambulation, (g) dexterity, and (h) cognition. Six attributes are obtained for the HUI2: (a) sensation, (b) mobility, (c) cognition, (d) self‐care, (e) emotion, and (f) pain. Attributes found in both the HUI2 and HUI3 include emotion, cognition and pain. These vary based on the following: (a) emotion in the HUI2 is based on anxiety whereas in the HUI3 it is based on happiness/unhappiness; (b) cognition in the HUI2 is based on learning and remembering whereas in the HUI3 it is based on forgetfulness and daily problem solving; and (c) pain in the HUI2 is based on the need for analgesics whereas in the HUI3 it is based on impairment of activities.^28^

Attribute levels are determined from responses provided for each multiple‐choice question or combination of questions according to an algorithm described previously^29^ and differ between the HUI2 and HUI3. Attribute levels represent a range of functional classes that categorize the level of disability using a noninterval scale. For the HUI2, attribute levels ranged from 1 to 4 (or 5), whereas for the HUI3 attribute levels ranged from 1 to 5 (or 6) (see Table 2). Attribute levels of 1 indicate normal/no impairment with increasing values reflecting increased impairment. The validity and reliability of the HUI system has been demonstrated in multiple languages, populations and across disease states.^27^, ^30^, ^31^, ^32^, ^33^, ^34^, ^35^, ^36^, ^37^, ^38^, ^39^

**Table 2:** Frequency and percent of HUI2 and HUI3 attribute levels as assessed by proxy and self

n (%)	Proxy						Self					
	Attribute level						Attribute level					
	1	2	3	4	5	6	1	2	3	4	5	6
HUI2												
Sensation	22 (34.4)	16 (25.0)	26 (40.6)	0	N/A	N/A	16 (40.0)	11 (27.5)	12 (30.0)	0	N/A	N/A
Mobility	41 (64.1)	16 (25.0)	5 (7.8)	2 (3.1)	0	N/A	28 (70.0)	6 (15.0)	3 (7.5)	2 (5.0)	0	N/A
Cognition	23 (35.9)	37 (57.8)	4 (6.3)	0	N/A	N/A	17 (42.5)	19 (47.5)	2 (5.0)	0	N/A	N/A
Self‐care	50 (78.1)	6 (9.4)	3 (4.7)	4 (6.3)	N/A	N/A	34 (85)	2 (5.0)	1 (2.5)	2 (5.0)	N/A	N/A
Emotion	38 (59.4)	23 (35.9)	3 (4.7)	0	0	N/A	22 (55.0)	10 (25.0)	6 (15.0)	1 (2.5)	0	N/A
Pain	47 (73.4)	10 (15.6)	6 (9.4)	0	0	N/A	27 (67.5)	11 (27.5)	0	0	0	N/A
HUI3												
Vision	40 (62.5)	23 (35.9)	0	0	1 (1.6)	0	23 (57.5)	16 (40.0)	1 (2.5)	0	0	0
Hearing	51 (79.7)	5 (7.8)	2 (3.1)	5 (7.8)	1 (1.6)	0	36 (90.0)	0	1 (2.5)	2 (5.0)	0	0
Speech	42 (65.6)	12 (18.8)	10 (15.6)	0	0	N/A	30 (75.0)	6 (15.0)	4 (10.0)	0	0	N/A
Cognition	23 (35.9)	10 (15.6)	5 (7.8)	22 (34.4)	4 (6.3)	0	17 (42.5)	4 (10.0)	4 (10.0)	11 (27.5)	2 (5.0)	0
Ambulation	42 (65.6)	15 (23.4)	2 (3.1)	3 (4.7)	1 (1.6)	1 (1.6)	30 (75.0)	5 (12.5)	2 (5.0)	1 (2.5)	1 (2.5)	1 (2.5)
Dexterity	41 (64.1)	19 (29.7)	1 (1.6)	2 (3.1)	1 (1.6)	0	31 (77.5)	6 (15.0)	0	1 (2.5)	1 (2.5)	0
Emotion	47 (73.4)	11 (17.2)	6 (9.4)	0	0	N/A	23 (57.5)	13 (32.5)	3 (7.5)	1 (2.5)	0	N/A
Pain	49 (76.6)	7 (10.9)	7 (10.9)	0	0	N/A	26 (65.0)	9 (22.5)	3 (7.5)	2 (5.0)	0	N/A

Note

For the HUI2 attribute levels are as follows in terms of disability: 1 = none; 2 = mild; 3 = moderate; and 4/5 = severe. For the HUI3, attribute levels differ based on the attribute. For vision, hearing and speech the attribute levels are as follows: 1 = none; 2 = mild, 3 and 4 = moderate; 5 and 6 = severe. For ambulation, dexterity, emotion, and pain the attribute levels are as follows: 1 = none; 2 = mild; 3 = moderate; 4, 5, and 6 = severe. Finally, for cognition the attribute levels are as follows: 1 = none, 2 and 3 = mild, 4 = moderate; 5 and 6 = severe.

Attribute levels are converted into single‐attribute utility scores^40^ that have interval scale properties ranging from 1.00 (no morbidity) to 0.00 (worst level of impairment). Single‐attribute utility scores can be combined to obtain a multi‐attribute utility function score (overall burden of morbidity) each for the HUI2 and HUI3. Multi‐attribute utility function scores have interval scale properties and range from 1.00 (no morbidity, perfect health) to 0.00 (dead). In order to receive a score of 1.00 (perfect health), the patient must have received a score of 1.00 at every attribute level. These utility scoring functions are based on published preference functions.^26^, ^27^, ^40^ Multi‐attribute utility function scores (ie overall burden of morbidity) were categorized such that a score of 1.00 indicated perfect health, a score of 0.89‐0.99 indicated mild burden of morbidity, a score of 0.70‐0.88 indicated moderate burden of morbidity and a score of <0.70 indicated severe burden of morbidity.

### 2.3 Molecular subgroup

Medulloblastoma samples were assigned subgroups by RNA NanoString technology, using the NanoString nCounter Analysis System at the University Health Network Microarray Centre. The content and methods have been described previously.^41^

### 2.4 Statistical analyses

#### 2.4.1 International sample

First, multi‐attribute utility function scores representing overall burden of morbidity of medulloblastoma were characterized as either perfect health (score of 1.00), mild (score of 0.89‐0.99), moderate (score of 0.70‐0.88), or severe (score of <0.70) for both the HUI2 and HUI3. We then calculated percentages for each burden of morbidity category. These percentages are reported separately for the self‐ and proxy‐assessed versions of the HUI.

#### 2.4.2 Regional comparisons

Both self‐assessed and proxy‐assessed scores were compared between sites from (a) Europe and North America (Western) versus (b) Asia (Eastern). Comparisons of all single‐attribute utility scores and multi‐attribute utility function scores (overall burden of morbidity) were conducted using Kruskal‐Wallis rank sum testing to determine if any regional differences existed in our sample. Since the HUI data is ordinal and not continuous, the Kruskal‐Wallis rank sum test was used. For this test, we calculated effect size using the epsilon‐squared—which indicates the degree to which one group has data with higher ranks than the other group. For epsilon‐squared, 0.01 to <0.08 indicates a small effect size, 0.08 to <0.26 is a medium effect size, and ≥0.26 is considered a large effect size.^42^

#### 2.4.3 Sample by subgroup

Distributions of all single‐attribute utility scores and multi‐attribute utility function scores (overall burden of morbidity) for the HUI2 and HUI3 as a function of subgroup were evaluated using Kruskal‐Wallis rank sum test. As with the *Regional comparisons*, effect sizes were calculated using epsilon‐squared where small effect sizes ranged from 0.01 to <0.08, medium effect sizes ranged from 0.08 to <0.26 and large effect sizes ranged from ≥0.26. Post hoc analyses of significant overall subgroup effects were performed using Dunn's Multiple Comparisons Test (False Discovery Rate *P* = .05) to determine specific subgroup differences.

#### 2.4.4 Sample by medical and demographic variables

The impact of relevant medical and demographic variables on single‐attribute utility scores and multi‐attribute utility function scores (overall burden of morbidity) was examined. Gender, metastatic status, extent of resection, treatment with CSI, and treatment with chemotherapy were compared using Kruskal‐Wallis rank sum test. As with the *Regional comparisons* and *Sample by subgroup*, effect sizes were calculated using epsilon‐squared where small effect sizes ranged from 0.01 to <0.08, medium effect sizes ranged from 0.08 to <0.26 and large effect sizes ranged from ≥0.26. Spearman Rank correlations were used to examine relations between single‐attribute utility scores and multi‐attribute utility function scores (overall burden of morbidity) with age at diagnosis and time since diagnosis.

The data that support the findings of this study are available on request from the corresponding author. The data are not publicly available due to privacy or ethical restrictions.

## 3 RESULTS

### 3.1 International sample

**Figure 1:**
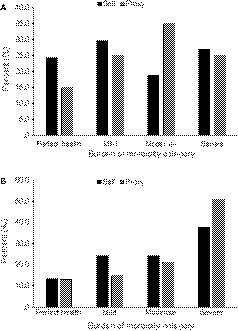
Percent overall burden of morbidity (ie a measure of the impact that medulloblastoma has on patients) reported using the (A) HUI2 and (B) HUI3 as assessed by self and proxy. Scores were categorized such that a score of 1.00 indicated perfect health, a score of 0.89‐0.99 indicated mild burden of morbidity, a score of 0.70‐0.88 indicated moderate burden of morbidity and a score of <0.70 indicated severe burden of morbidity

Percent burden of morbidity assessed by self and proxy as determined by the HUI2 and HUI3 are displayed in Figure 1A,B, respectively. For the proxy‐assessed scores, the majority of patients were rated as having moderate or severe overall burden of morbidity for both the HUI2 and HUI3 (HUI2 = 60%; HUI3 = 72.1%). For both the HUI2 and HUI3, the least frequent rating for proxy‐assessed scores was “perfect health” (HUI2 = 15%; HUI3 = 13.1%). In contrast, fewer patients reported moderate or severe overall burden of morbidity for the HUI2 (45.9%), but not the HUI3 (62.1%) based on self‐assessed scores, and more reported “perfect health” or mild burden of morbidity on the HUI2 (54%) when self‐assessed versus 40% when proxy assessed. Frequencies and percentages for each attribute level for both proxy‐ and self‐assessed scores on the HUI2 and HUI3 are displayed in Table 2.

To maximize our sample size for the further analysis of region, subgroup, and medical and demographic variables, proxy‐assessed scores were combined with self‐assessed scores when only the latter were available (n = 76). We note that there were no significant differences in any single‐attribute utility scores for the proxy‐ and self‐assessed versions in participants where both versions were acquired (*P* > .05). Furthermore, the distribution of overall burden of morbidity and attribution levels were similar for this combined proxy/self‐assessed sample as compared to proxy alone.

### 3.2 Regional comparisons

Means, standard deviations, effect sizes (epsilon‐squared), and *P*‐values of single‐attribute utility scores and multi‐attribute utility function scores (overall burden of morbidity) for Western versus Eastern sites are displayed in Table 3. Analyses revealed a statistically significant difference in the proxy‐assessed single‐attribute utility score for self‐care on the HUI2 with a distribution of higher rank indicating better outcomes, observed for the Western versus Eastern sites (H(1) = 5.280, Ɛ^2^ = 0.085, *P* = .02). No other statistically significant differences were found among the other single‐attribute utility scores as a function of region when assessed by either proxy or self. In regards to overall burden of morbidity, no significant regional differences were found with either the HUI2 or HUI3.

**Table 3:** HUI2 and HUI3 single‐attribute utility scores and multi‐attribute utility function scores (ie overall burden of morbidity) as a function of region when assessed by proxy and self

Site	Proxy						Self					
	Western		Eastern		ε^2^	*P*‐value	Western		Eastern		ε^2^	*P*‐value
	Mean	SD	Mean	SD			Mean	SD	Mean	SD		
HUI2												
Sensation	0.929	0.06	0.934	0.06	0.000	.88	0.939	0.06	0.946	0.06	0.007	.61
Mobility	0.972	0.06	0.969	0.07	0.000	.93	0.977	0.05	0.961	0.09	0.003	.75
Cognition	0.96	0.03	0.98	0.03	0.035	.14	0.964	0.04	0.973	0.03	0.006	.63
Self‐care	0.98	0.05	0.97	0.05	0.085	.02*	0.999	0.01	0.973	0.06	0.061	.12
Emotion	0.96	0.05	0.97	0.05	0.017	.3	0.939	0.08	0.952	0.08	0.005	.66
Pain	0.98	0.51	0.99	0.01	0.004	.62	0.991	0.01	0.992	0.01	0.003	.72
Overall	0.81	0.15	0.83	0.17	0.002	.71	0.824	0.18	0.809	0.19	0.002	.81
HUI3												
Vision	0.99	0.04	0.99	0.01	0.011	.42	0.99	0.01	0.989	0.02	0.016	.43
Hearing	0.965	0.07	0.994	0.03	0.049	.08	0.979	0.06	0.995	0.02	0.013	.48
Speech	0.97	0.04	0.97	0.05	0.010	.44	0.983	0.03	0.978	0.04	0.001	.86
Cognition	0.89	0.11	0.93	0.08	0.021	.25	0.901	0.13	0.931	0.08	0.005	.68
Ambulation	0.96	0.08	0.95	0.11	0.000	.91	0.973	0.07	0.944	0.12	0.015	.44
Dexterity	0.97	0.05	0.96	0.09	0.000	.93	0.977	0.06	0.978	0.08	0.016	.42
Emotion	0.98	0.04	0.97	0.06	0.000	.94	0.952	0.09	0.975	0.04	0.008	.58
Pain	0.98	0.04	0.99	0.03	0.0245	.22	0.968	0.06	0.977	0.05	0.002	.77
Overall	0.67	0.25	0.72	0.31	0.011	.41	0.73	0.32	0.715	0.27	0.006	.62

Note

Single‐attribute utility scores have interval scale properties ranging from 1.00 (no morbidity) to 0.00 (worst level of impairment). For overall burden of morbidity (multi‐attribute utility function scores) a score of 1.00 indicates perfect health, a score of 0.89‐0.99 indicates mild burden of morbidity, a score of 0.70‐0.88 indicates moderate burden of morbidity and a score of <0.70 indicated severe burden of morbidity. Epsilon‐squared (ε^2^) effect sizes: small = 0.01 to <0.08, medium = 0.08 to <0.26, and large = ≥0.26.

Abbreviations: ε^2^, epsilon‐squared; SD, standard deviation.

* Indicates a statistically significant difference (*P* ≤ .05).

### 3.3 Sample by subgroup

Kruskal‐Wallis rank sum test results for comparisons of subgroups with single‐attribute utility scores and multi‐attribute utility function scores (overall burden of morbidity) are presented in Table 4. No statistically significant results were found.

**Table 4:** Differences between HUI2 and HUI3 single‐attribute utility scores and multi‐attribute utility function scores (ie overall burden of morbidity) as a function of subgroup, demographic, and medical variables

	Subgroup			Gender			Metastatic status			CSI			Resection			Chemotherapy		
	χ^2^	ε^2^	*P*‐value	χ^2^	ε^2^	*P*‐value	χ^2^	ε^2^	*P*‐value	χ^2^	ε^2^	*P*‐value	χ^2^	ε^2^	*P*‐value	χ^2^	ε^2^	*P*‐value
HUI2																		
Sensation	2.42	0.032	.49	0.37	0.005	.55	0	0.000	.96	0.822	0.011	.66	0.415	0.006	.52	1.56	0.021	.21
Mobility	4.56	0.061	.21	1.09	0.015	.30	0.02	0.000	.90	0.81	0.011	.67	0.014	0.000	.91	0.51	0.007	.48
Cognition	4.46	0.060	.22	0.26	0.003	.61	1.26	0.017	.26	3.149	0.042	.21	1.735	0.023	.19	1.61	0.021	.20
Self‐care	3.3	0.044	.35	0.000	0.000	.99	1.26	0.017	.26	1.64	0.022	.44	0.003	0.000	.95	0.230	0.003	.63
Emotion	1.27	0.017	.74	2.51	0.033	.11	1.18	0.016	.28	3.102	0.041	.21	1.363	0.018	.24	0.71	0.010	.40
Pain	5.42	0.072	.14	0.01	0.000	.95	3.98	0.053	.05*	1.190	0.016	.55	1.327	0.018	.25	0.39	0.005	.53
Overall	2.37	0.032	.50	0.01	0.000	.91	1.03	0.014	.31	1.194	0.016	.55	0.096	0.001	.76	2.17	0.029	.14
HUI3																		
Vision	1.37	0.018	.71	1.240	0.017	.27	0.66	0.009	.42	0.369	0.005	.83	5.230	0.070	.02*	0.7	0.009	.40
Hearing	4.730	0.063	.19	0.01	0.000	.92	4.59	0.061	.03*	2.138	0.029	.34	4.150	0.055	.04*	0.21	0.003	.65
Speech	0.55	0.007	.91	0	0.000	.95	0.11	0.001	.74	1.162	0.016	.56	0.627	0.008	.43	0.45	0.006	.50
Cognition	3.03	0.040	.39	0.26	0.003	.61	0.5	0.007	.48	3.046	0.041	.22	0.830	0.011	.36	1.36	0.018	.24
Ambulation	4.480	0.060	.21	0.73	0.010	.39	0.06	0.001	.80	0.594	0.008	.74	0.067	0.001	.80	0.48	0.006	.49
Dexterity	0.03	0.000	1.00	0.39	0.005	.53	0.09	0.001	.76	2.067	0.028	.36	0.220	0.003	.64	1.77	0.024	.18
Emotion	3.26	0.043	.35	1.81	0.024	.18	0	0.000	.96	3.446	0.046	.18	0.000	0.000	1.00	0.46	0.006	.50
Pain	2.47	0.033	.48	0.38	0.005	.54	4.81	0.064	.03*	0.890	0.012	.64	0.211	0.003	.65	0.36	0.005	.55
Overall	2.15	0.029	.54	0.06	0.001	.81	0.7	0.009	.40	0.775	0.010	.68	0.423	0.006	.52	0.92	0.012	.34

Note

Single‐attribute utility scores have interval scale properties ranging from 1.00 (no morbidity) to 0.00 (worst level of impairment). For overall burden of morbidity (multi‐attribute utility function scores) a score of 1.00 indicates perfect health, a score of 0.89‐0.99 indicates mild burden of morbidity, a score of 0.70‐0.88 indicates moderate burden of morbidity and a score of <0.70 indicated severe burden of morbidity. Epsilon‐squared (ε^2^) effect sizes: small = 0.01 to <0.08, medium = 0.08 to <0.26, and large = ≥0.26.

Abbreviations: CSI, craniospinal irradiation; ε^2^, epsilon‐squared; χ^2^, chi‐square.

* Indicates a statistically significant difference (*P* ≤ .05).

### 3.4 Sample by medical and demographic variables

**Figure 2:**
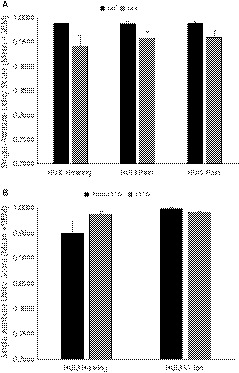
Significant findings (*P* < .05) for single‐attribute utility scores from the HUI2 and HUI3 across the entire sample in relation to (A) metastatic status and (B) resection. Single‐attribute utility scores range from 1.00 (no morbidity) to 0.00 (worst level of impairment). Mean values and standard error of the mean (SEM) are shown. GTR, gross total resection; M0, nonmetastatic; M+, metastatic

Kruskal‐Wallis rank sum test results for comparisons of medical and demographic variables with single‐attribute utility scores and multi‐attribute utility function scores (overall burden of morbidity) are presented in Table 4. Patients with nonmetastatic status presented with higher values (ie better outcomes) for HUI3 hearing (H(1) = 4.594, Ɛ^2^ = 0.061, *P* = .03), HUI3 pain (H(1) = 4.806, Ɛ^2^ = 0.064, *P* = .03) and HUI2 pain (H(1) = 3.976, Ɛ^2^ = 0.053, *P* = .05) than patients with a positive metastatic status (Figure 2A). Further, patients treated with a gross total resection had the highest values (ie better outcomes) for HUI3 hearing (H(1) = 4.150, Ɛ^2^ = 0.055, *P* = .04) and lowest values (ie worse outcomes) for the HUI3 vision (H(1) = 5.230, Ɛ^2^ = 0.070, *P* = .02) (Figure 2B). No other statistically significant results were found. Finally, we observed a relation between time since diagnosis and HUI3 vision (Spearman Rank correlation *P *= −.256) suggesting that as survivors continue to grow and develop, vision problems worsen. No other correlations were found between the single‐attribute utility scores and multi‐attribute utility function scores (overall burden of morbidity) and age at diagnosis or time since diagnosis (Spearman Rank correlations *P* > .05).

## 4 DISCUSSION

Here we expand the sparse body of literature examining HRQL in large multi‐site cohorts of pediatric medulloblastoma survivors.^43^ Although medulloblastoma is the most common malignant brain tumor among children worldwide the overall incidence of this disease is low (0.49 per 100 000 children per year^44^). Consequently, obtaining both tissue samples and outcome data in large cohorts of medulloblastoma patients is challenging. Most studies of HRQL are limited to geographically homogeneous cohorts^45^ or heterogeneous brain tumor diagnoses.^14^, ^15^ We show, for the first time, that HRQL is compromised in an international multi‐continental sample of pediatric medulloblastoma survivors, where the majority of patients reported moderate or severe overall burden of morbidity following treatment when assessed by proxy. However, when patients completed the HUI, they reported less moderate to severe burden of morbidity on the HUI2, but not the HUI3. This finding suggests that pediatric brain tumor survivors do not interpret their abilities as burdensome as their caregivers. Caregiver expectations may be greater and since they are responsible for supporting and caring for the patients, they may compensate for some of the deficits the children have without the children realizing them.

In regards to HRQL according to geographic location (ie, North American and European versus Asian sites), we only observed a difference in HUI2 self‐care such that patients from Western sites reported better performance compared to those from Eastern sites, but only when assessed by proxy. Studies examining social competence in pediatric brain tumor survivors in Canada revealed that only patients diagnosed with medulloblastoma were found to have lower self‐report ratings of social competence.^46^ This finding was purported to be associated with impairments in cognition and independent living, which have previously been reported.^47^, ^48^

Overall, HRQL does not appear to be influenced by geographic factors in our international cohort. Our findings reinforce the global impact of this disease and the need for better understanding of current treatments on HRQL in not only Western, but also Eastern sites. As a global health issue, emphasis on international collaboration is required to reduce the burden of morbidity of this disease.

When individual medical variables were used to analyze outcome measures, we observed that patients with metastatic disease expressed worse hearing and pain outcomes. Typical standard of care for children with a positive metastatic status involves treatment with higher doses of CSI than those with nonmetastatic disease and CSI is typically associated with poorer hearing outcomes.^49^, ^50^, ^51^ Extent of resection was also found to have an impact on some single‐attribute utility scores. Children who underwent a gross total resection reported having better scores for hearing. Perhaps the successful surgical removal of the tumor minimized the amount of damage to the brain caused by any remaining tumor and/or resulted in reduced subsequent treatment intensity required to treat the residual tumor, similar to what is required for the metastatic disease outcomes described above. However, children who received a gross total resection reported worse vision scores. Medulloblastoma tumors are located in the posterior fossa, which is near the occipital lobe which is important for vision. During a gross total resection, perhaps some healthy tissue is damaged resulting in visual deficits.

Finally, when we correlated all single‐attribute utility scores and multi‐attribute utility function scores (overall burden of morbidity) with age at diagnosis and time since diagnosis, we only found a negative correlation between time since diagnosis and vision—as time since diagnosis increases, pediatric medulloblastoma survivors reported having worse vision. In comparison to other pediatric brain tumors, medulloblastoma is associated with having one of the worst prognosis for vision outcomes (poor or fair).^52^ Vision problems are one of the late effects reported in medulloblastoma survivors.^14^ The negative impact on vision in medulloblastoma survivors is not surprising as primary vision brain structures are located at the back of the brain, near the area receiving the most treatment.

By examining a large international sample of survivors, we can characterize the impact of medulloblastoma worldwide. This is a critical first step in developing standards for evaluating long‐term outcomes in survivors of pediatric medulloblastoma. Since prognosis varies by subgroup (ie WNT good prognosis, Group 3 poor prognosis), determining HRQL posttreatment is important for refining current treatment protocols. This is especially important when we consider outcome differences between high and low to middle income countries. High income countries report incidence rates of 20%‐25%, whereas low to middle income countries report 6.1%‐49.4%.^1^ Diagnostic and treatment protocols vary across the world due to—among other things—financial and logistic factors.^7^, ^53^ Ultimately, these inconsistencies can result in a disparity of survival rates and HRQL.^54^ As such, it would be interesting to see if our results would differ if low to middle income countries were included. Future studies exploring HRQL in medulloblastoma survivors using international samples could further analyze the impact of those who live in high vs. low to middle income countries. Initiatives are underway to improve outcomes globally—for example, the SIOP Pediatric Oncology in Developing Countries group (PODC‐SIOP) has made recommendations for treating medulloblastoma in low to middle income countries, some of which include surgical techniques, timing and planning of CSI, and surveillance of late effects to help improve HRQL.^1^ While the focus on such initiatives is survival, as outcomes improve we think it is equally important to consider HRQL in all childhood survivors of medulloblastoma—no matter their country of origin.

Limitations of this study include the use of a single measure of HRQL. Other validated questionnaires that have been translated into other languages, such as the World Health Organization WHOQOL‐100 or WHOQOL‐BREF or Pediatric Quality of Life Inventory, should be used to help verify our findings. Despite using an international sample, our cohort included patients predominately treated in North America and had few WNT patients. This was not surprising as WNT is the rarest subgroup, further emphasizing the need for multi‐site collaboration. Despite this limitation, we do note that ours is the only cohort we are aware of that includes patients from Europe, Asia, and North America. However, we do not know the representativeness of our sample, since we do not have specific numbers regarding those who chose to participate and those who refused, potentially leading to selection bias. Future studies should further characterize the sample population by including details regarding the presence of hydrocephalus, cerebellar mutism, hormone deficiency, etc. In addition, we note that although our study combined proxy‐ and self‐assessed versions, efforts should be made to obtain consistent respondent types, either proxy‐assessed, self‐assessed or both, in future studies using the HUI. Given the exploratory nature of this study, we did not correct for multiple comparisons, therefore findings may reflect the result of Type I error. HRQL was only assessed at one time point. As late effects continue to develop into widespread deficits, it would be beneficial to assess HRQL over time. These results could be used to plan long‐term follow‐up services and initiate potential preventative measures. Our sample did not include any survivors from low to middle income countries. Since evidence shows a disparity between high and low to middle income countries, it is important to include patients from these countries when assessing HRQL in survivors.

Despite the significant advances in our knowledge of medulloblastoma and the resulting advantages of improved therapy, enhanced HRQL has not been realized globally. Promoting international collaboration with the incorporation of a standardized measure of HRQL is vital for improving patient outcomes for every child.

## Data Availability

The data that support the findings of this study are available on request from the corresponding author. The data are not publicly available due to privacy or ethical restrictions.
